# Nurses’ well-being and its relationship with quality of nursing work life at Alsalam and Al Haram Hospitals, Saudi Arabia

**DOI:** 10.3389/fpubh.2025.1678358

**Published:** 2025-10-29

**Authors:** Meshal S. Alharbi, Mohammed B. Albughuli, Sami S. Aljohani, Yahya A. Alraddadi, Najeeb B. Alalawi, Hani M. Alawfi, Sami M. Al Harbi, Rasha H. Alharbi, Ohoud A. Alreshidi

**Affiliations:** ^1^Department of Nursing Education, Al-Salam Hospital, Ministry of Health, Al-Madinah Region, Saudi Arabia; ^2^Department of Nursing Office, Al-Salam Hospital, Ministry of Health, Al-Madinah Region, Saudi Arabia; ^3^Department of Nursing Supervisors, Al-Salam Hospital, Ministry of Health, Al-Madinah Region, Saudi Arabia; ^4^Department of Intensive Care (ICU), Al-Salam Hospital, Ministry of Health, Al-Madinah Region, Saudi Arabia; ^5^Medical Department, Al-Salam Hospital, Ministry of Health, Al-Madinah Region, Saudi Arabia; ^6^Surgical Department, King Salman Specialist Hospital, Ministry of Health, Hail Region, Saudi Arabia

**Keywords:** nurses, work environment, quality of life, Saudi Arabia, occupational well-being

## Abstract

**Introduction:**

Nurses’ well-being is a vital factor influencing healthcare quality, patient safety, and workforce sustainability. In Saudi Arabia, nurses in public hospitals often encounter high workloads, emotional stress, and limited managerial support, potentially affecting both their mental health and professional performance.

**Aims:**

This study aimed to assess the well-being of nurses working at Alsalam and Al Haram hospitals in Saudi Arabia and to examine its relationship with the Quality of Nursing Work Life (QNWL).

**Methods:**

A cross-sectional census study was conducted in 2025 involving 169 registered nurses from the two hospitals. Data were collected using structured interview questionnaires incorporating three validated tools: The Nurse’s Well-Being Assessment Tool (NWAT), the World Health Organization-Five Well-Being Index (WHO-5), and the QNWL Scale. Statistical analysis was performed using SPSS version 25.

**Results:**

Nurses reported moderate overall well-being (mean NWAT score: 62.20 ± 2.9), with notably low scores in work-life balance and emotional/mental health. The WHO-5 index indicated low emotional well-being (mean: 12.70 ± 2.2), reflecting significant emotional distress. The QNWL scores were generally poor (mean: 91.02 ± 6.0), particularly in the areas of managerial support and work-life balance. Moderate scores were observed in nurse-physician relationships and professional fulfillment. A significant positive correlation was found between well-being (NWAT and WHO-5) and QNWL scores (*p* = 0.001), indicating that higher well-being is associated with better quality of work life. Comparisons between hospitals showed that nurses at Alsalam Hospital had higher QNWL and WHO-5 scores but lower NWAT scores than those at Al Haram Hospital, with statistically significant differences (*p* < 0.05).

**Conclusion:**

Nurses at both hospitals face multiple well-being challenges that negatively impact their quality of work life. Interventions focusing on mental health support, enhanced managerial engagement, and improved work-life balance are essential. These efforts are crucial for advancing workforce well-being and aligning with the healthcare objectives of Saudi Arabia’s Vision 2030.

## 1 Introduction

Nurses are the backbone of healthcare systems worldwide, providing continuous, patient-centered care that directly impacts clinical outcomes, safety, and overall patient satisfaction (1). In an era of rising healthcare demands, workforce shortages, and increasing patient complexity, the well-being of nurses has emerged as a critical determinant of healthcare quality and sustainability (2). Globally, nurse well-being is increasingly recognized as essential not only for individual health but also for organizational performance, quality of care, and healthcare system resilience (3).

In Saudi Arabia, the urgency of addressing nurse well-being is compounded by ongoing healthcare reforms under Vision 2030, a strategic initiative that seeks to enhance service efficiency, improve patient access, and reduce reliance on foreign healthcare professionals (4). While these reforms have driven significant advancements in infrastructure, digital transformation, and medical services, human resource development especially regarding frontline nursing staff, remains under-emphasized (5).

Nurses in Saudi public hospitals often contend with high workloads, emotional strain, inconsistent managerial support, and extended shifts, all of which contribute to burnout, job dissatisfaction, and reduced well-being (6). A substantial portion of the nursing workforce is made up of expatriates, who face additional psychosocial stressors including cultural dissonance, language barriers, limited social support, and marginalization, further impacting their Quality of Nursing Work Life (QNWL) (7).

Well-being among nurses is a multidimensional construct, encompassing emotional, psychological, social, and physical domains (8). In practice, low well-being among nurses is associated with increased absenteeism, poor interpersonal relationships, diminished clinical performance, and elevated turnover rates, all of which compromise the overall quality of healthcare services (9). QNWL, as a framework, integrates nurses’ satisfaction with the work environment, interpersonal dynamics, autonomy, support systems, and their ability to maintain a work-life balance (10). Thus, the link between well-being and QNWL is both logical and critical to explore particularly in complex healthcare settings like those found in Saudi Arabia.

Despite increasing awareness, empirical data on the relationship between nurses’ well-being and QNWL in Saudi Arabia remains limited, especially in public, secondary-level hospitals such as Alsalam and Al Haram. Previous studies have primarily focused on either job satisfaction or burnout, often neglecting comprehensive well-being measures or contextual workplace variables (6, 11). This gap in localized evidence hinders the development of effective policies aimed at nurse retention, mental health support, and sustainable workforce planning (7).

This study seeks to address that gap by examining the well-being of nurses at Alsalam and Al Haram hospitals and its association with their perceived QNWL. By using validated tools such as the Nurse’s Well-Being Assessment Tool (NWAT) (12), WHO-5 Well-Being Index (13), and the QNWL scale (14), this research offers a holistic understanding of the nurses’ experience. Furthermore, it provides comparative insight between two public hospitals facing similar systemic pressures but potentially differing in workplace culture and managerial practices.

As Saudi Arabia continues its transformation toward a knowledge-based, high-quality healthcare system, ensuring the psychological and professional sustainability of its nursing workforce is indispensable. This study aims to provide actionable insights for healthcare administrators, policymakers, and organizational leaders to develop targeted interventions that enhance nurses’ well-being, strengthen QNWL, and ultimately improve patient care and organizational outcomes in alignment with Vision 2030 goals (4, 5).

## 2 Materials and methods

### 2.1 Study design

This study adopted a cross-sectional design to evaluate nurses’ well-being and its relationship with the QNWL at Alsalam and Al Haram hospitals in Saudi Arabia. By collecting data at one point in time, it provided a clear snapshot of current conditions and efficiently examined the associations between key factors without the need for long-term observation or experimental methods.

### 2.2 Study setting

The study was conducted at Alsalam and Al Haram hospitals, prominent public healthcare institutions in Al-Madina El Monawara, Saudi Arabia. These multidisciplinary hospitals offered a wide range of services, including emergency, medical, and intensive care. Together, they had a total capacity of 152 beds (81 at Alsalam and 71 at Al Haram) and served both inpatients and outpatients. The healthcare team included nurses, physicians, technicians, and administrative staff who collaborated to deliver comprehensive care. Nurses played particularly vital in high-demand areas like intensive care and emergency services. The hospitals were equipped with modern infrastructure, advanced medical technology, and support services that enhanced patient care and outcomes (15).

### 2.3 Study period

The study was conducted over a four-month period, from March to July 2025.

### 2.4 Study population

The study population consisted of all registered nurses employed at Alsalam and Al Haram hospitals in Al-Madina El Monawara, Saudi Arabia, encompassing those working in a variety of departments.

### 2.5 Inclusion criteria

The inclusion criteria for the study were: registered nurses currently employed at Alsalam and Al Haram hospitals with a minimum of 6 months of work experience to ensure adequate familiarity with the hospital setting. Eligible participants included those working in hospital units, supervisors, and those in the nursing office. Participation was voluntary and required informed consent.

### 2.6 Exclusion criteria

The exclusion criteria for the study included nurses who were on extended leave (such as maternity, medical, or vacation leave) during the data collection period. Interns, nursing students, and trainees who were not registered nurses were also excluded. Furthermore, nurses who declined to participate or submitted incomplete or improperly filled questionnaires were not included in the study.

### 2.7 Sample size and sampling technique

A census sampling approach was employed to meet the study’s objective of comprehensively evaluating nurses’ well-being and QNWL at Alsalam and Al Haram hospitals. This method involved the complete enumeration of all 169 eligible registered nurses (108 at Alsalam Hospital and 61 at Al Haram Hospital) working across clinical and administrative departments during the study period. This approach minimized sampling bias and allowed for a full, representative assessment of the institutional nursing workforce, which is especially appropriate when addressing organization-wide issues such as well-being and work-life quality (16). A census sampling approach was used, wherein all 169 eligible registered nurses employed at Alsalam and Al Haram hospitals during the study period were invited to participate. All 169 nurses completed the survey, resulting in a response rate of 100%. While uncommon, this rate is explained by several contextual and procedural factors. First, data collection was carried out by trained researchers independent of hospital administration, ensuring neutrality and minimizing perceived coercion. Second, informed consent was obtained from all participants, who were assured of the anonymity and confidentiality of their responses, and were explicitly informed that participation was voluntary with no professional repercussions. No incentives were offered. Third, the nursing workforce at both hospitals is relatively small and cohesive, and the study’s focus on nurses’ well-being and work-life quality, issues of high personal and professional relevance likely enhanced willingness to participate. Lastly, the supportive organizational culture and leadership encouragement to engage in quality and safety initiatives may have further contributed to the strong participation rate.

### 2.8 Data collection

To effectively achieve the study objectives, data were collected using a structured, interview-based questionnaire that incorporated the following validated and reliable tools:

#### 2.8.1 Part I: demographic and socioeconomic questions

A brief questionnaire was used to gather demographic and socioeconomic information from nurses, including age and biological sex (male/female), marital status, years of experience, department, education level, income level, nationality and other relevant factors.

#### 2.8.2 Part II: nurse’s well-being assessment tool (NWAT)

The NWAT is a structured self-assessment instrument designed to evaluate nurses’ overall well-being across several key domains. It helps healthcare organizations identify factors affecting job satisfaction, stress, and professional performance. The tool systematically assesses physical, emotional, and professional aspects of well-being to guide targeted interventions aimed at improving work environments, promoting resilience, and enhancing nurse retention. The domains covered include: (1) Physical Well-being: Evaluates energy levels, sleep quality, and physical health; (2) Emotional and Mental Health: Measures stress levels, coping abilities, and emotional support; (3) Workload and Burnout: Assesses perceptions of overwork, emotional exhaustion, and burnout symptoms; (4) Work Environment & Support: Covers workplace safety, respect, feedback, and teamwork; (5) Work-Life Balance: Focuses on boundaries between work and personal life; and (6) Professional Fulfilment: Assesses job satisfaction, purpose, and development opportunities (12).

##### 2.8.2.1 Scoring and interpretation

Each item in the NWAT is scored on a scale from 1 to 5, with higher scores indicating greater well-being. The total score ranges from 18 (low well-being) to 90 (high well-being). The interpretation of scores is as follows: (1) 76–90: High well-being, maintain current strategies, (2) 60–75: Moderate well-being, some areas need support, and (3) <60: Low well-being, requires targeted intervention (12).

#### 2.8.3 Part III: the World Health Organization-5 well-being index (WHO-5)

The World Health Organization-5 Well-Being Index (WHO-5) is an ideal tool for assessing the emotional well-being of nurses at Alsalam and Al Haram hospitals. Its short length, proven validity, and cross-cultural relevance make it well-suited for quickly evaluating mental health and gaining insights into factors affecting nurses’ QNWL (13). The WHO-5 Well-Being Index is a brief, self-reported tool designed to assess an individual’s emotional well-being over the past 2 weeks. Developed by the World Health Organization in the 1990s, it has been widely used across diverse populations and clinical settings to measure general psychological well-being and mental health. Renowned for its reliability, validity, and simplicity, the WHO-5 is an effective instrument for quickly screening and monitoring well-being in both general and clinical groups (13). The WHO-5 consists of five positively phrased items that assess mood, energy, and overall emotional well-being, such as feeling cheerful, calm, active, rested, and interested in daily life. Respondents rate each statement based on their experiences over the past 2 weeks using a 6-point Likert scale ranging from 0 (at no time) to 5 (all of the time). This simple and effective tool captures key aspects of emotional health in a concise format.

##### 2.8.3.1 Scoring and interpretation

Responses to the five WHO-5 items are summed and converted to a total score ranging from 0 to 25, with higher scores indicating better emotional well-being. Scores above 13 generally reflect good well-being, while scores below 13 may signal low well-being and the need for further assessment or support, particularly concerning mental health issues like depression or anxiety (13).

#### 2.8.4 Part IV: quality of nursing work life (QNWL) scale

The QNWL Scale developed by Brooks and Anderson (14). The questions are designed to assess multiple aspects of a nurse’s work environment, including work-life balance, relationships with physicians, managerial support, workload, and job satisfaction. The QNWL Scale provides a comprehensive way to evaluate nurses’ experiences across these key areas. Using this tool, the study can gain valuable insights into factors affecting nurses’ well-being and job satisfaction, helping to identify areas needing intervention to reduce burnout, improve job satisfaction, and enhance the quality of nursing care at Alsalam and Al Haram hospitals.

##### 2.8.4.1 Scoring the QNWL scale and Interpretation of scores

After the survey is completed, scores for each of the six dimensions, work-life balance, nurse-physician relationships, managerial support, communication and interaction, workload and work stress, and autonomy and professional fulfilment are summed. The total score, ranging from 34 to 170 points, reflects the overall quality of nursing work life, with higher scores indicating better quality (17, 18). Interpretation of scores: Scores above 130 indicate a high quality of nursing work life, reflecting a well-supported and satisfying work environment for nurses. Scores between 100 and 130 represent a moderate quality of work life, where some areas may require improvement but overall conditions are acceptable. Scores below 100 suggest a poor quality of work life, signalling the need for significant enhancements in the work environment, workload management, or organizational support (17, 18).

### 2.9 Pilot study

A pilot study was conducted with 20 nurses to evaluate the study tools. Based on the findings, the questionnaire and data collection procedures were revised to improve clarity and effectiveness.

### 2.10 The reliability and validity of the study tools

The NWAT, WHO-5 Index, and QNWL Scale are essential tools for accurately evaluating nurses’ well-being and quality of work life at Alsalam and Al Haram hospitals. These instruments have shown strong reliability in prior studies, with high Cronbach’s alpha values ranging from 0.85 to 0.92 for the QNWL and 0.85 to 0.90 for the WHO-5, indicating excellent internal consistency (19, 20). To verify their reliability in this specific setting, a pilot study was conducted with 20 nurses. This allowed for the assessment of internal consistency using Cronbach’s alpha and test–retest reliability by administering the tools twice at different times. Any inconsistencies found were addressed and corrected before the main study. Validity was established through expert evaluations to ensure content validity, confirming that the instruments thoroughly cover relevant dimensions of well-being and work-life quality. Construct validity was also assessed to ensure the tools accurately measure the intended concepts, while pilot testing helped identify and resolve any issues with wording, relevance, or cultural appropriateness. Additionally, criterion-related validity was evaluated by comparing the instruments’ outcomes with other established measures, further confirming their suitability for this study population. These combined steps ensured that the tools used are both reliable and valid for assessing nurses’ well-being and work-life quality at Alsalam and Al Haram hospitals.

### 2.11 Statistical analysis

Statistical analysis was performed using the Statistical Package for Social Sciences (SPSS) version 25. Data are expressed as means ± standard deviations (SD) for continuous variables and as percentages for categorical variables. Differences between means were tested using the Independent-Samples T Test and One-Way ANOVA. The chi-square test was used to examine differences in the prevalence of categorical variables. A *p*-value of less than 0.05 was considered statistically significant.

## 3 Results

A total of 169 nurses participated in the final analysis, with 108 (63.9%) from Alsalam Hospital and 61 (36.1%) from Al Haram Hospital. Among them, 59 (35.0%) were male and 110 (65.0%) were female. The average age of participants was 36.68 ± 8.1 years. A majority, 112 (66.3%), were 40 years old or younger. Regarding marital status, 140 (82.8%) were married. More than half of the nurses, 92 (54.4%), held a bachelor’s degree in nursing, while only 5 (3.0%) had a master’s degree. The average work experience was 3.42 ± 3.2 years, with 139 (82.2%) having more than 1 year of experience. In terms of department distribution, 63 (37.3%) worked in the emergency room, 48 (28.4%) in the intensive care unit, 47 (27.8%) in the medical department, and 11 (6.5%) served as nursing supervisors in the nursing office. All participating nurses reported receiving job-related orientation training upon employment, with an average duration of 2.68 ± 1.3 weeks. Notably, 122 nurses (72.2%) reported completing more than 1 week of orientation. The mean monthly income was 11,198.81 ± 83.7 Saudi Riyals, with the vast majority, 162 (95.9%), earning more than 10,000 Saudi Riyals per month. Most participants were Saudi nationals (135; 79.9%), while 34 (20.1%) were non-Saudi. Statistically significant differences were observed between the two hospitals in terms of age categories, education level, years of experience, and monthly income (all with *p*-values < 0.05), as detailed in Table 1.

**Table 1:** Characteristics of the study participants.

Variables	Total *N* = 169 (100%)	Alsalam hospital *N* = 108 (63.9%)	Al Haram hospitals *N* = 61 (36.1%)	*p* values
Age (years), mean ± SD: 36.68 ± 8.1				
Age categories				
≤ 40 years	112 (66.3)	77 (71.3)	35 (57.4)	0.048
More than 40 years	57 (33.7)	31 (28.7)	26 (42.6)	
Sex				
Male	59 (35.0)	38 (35.2)	21 (34.4)	0.529
Female	110 (65.0)	70 (64.8)	40 (65.6)	
Marital status				
Single	29 (17.2)	18 (16.7)	11 (18.0)	0.489
Married	140 (82.8)	90 (83.3)	50 (82.0)	
Education level				
Diploma	72 (42.6)	38 (35.2)	34 (55.7)	0.014
Bachelor	92 (54.4)	65 (60.2)	27 (44.3)	
Master	5.0 (3.0)	5.0 (4.6)	0.0 (0.0)	
Years of experience, mean ± SD: 3.42 ± 3.2				
Years of experience categories				
≤1 ear	30 (17.8)	30 (27.8)	0.0 (0.0)	0.001
More than 1 year	139 (82.2)	78 (72.2)	61 (100)	
Department				
Emergency room	63 (37.3)	37 (34.3)	26 (42.6)	0.532
Intensive care unit	48 (28.4)	34 (31.5)	14 (23.0)	
Medical department	47 (27.8)	29 (26.9)	18 (29.5)	
Supervisors (Nursing office)	11 (6.5)	8.0 (7.3)	3.0 (4.9)	
Received job-related orientation training				
Yes	169 (100)	108 (63.9)	61 (36.1)	-
Job-related orientation training duration (week), mean ± SD: 2.68 ± 1.3				
Job-related orientation training duration categories				
≤ one week	47 (27.8)	29 (26.9)	18 (29.5)	0.421
More than one week	122 (72.2)	79 (73.1)	43 (70.5)	
Monthly income (Saudi Riyal), mean ± SD: 1198.81 ± 83.7				
Monthly income categories				
Less than 10000 Saudi Riyal	7.0 (4.1)	7.0 (6.5)	0.0 (0.0)	0.040
≥10000 Saudi Riyal	162 (95.9)	101 (93.5)	61 (100)	
Nationality				
Saudi	135 (79.9)	85 (78.7)	50 (82.0)	383
Non-Saudi	34 (20.1)	23 (21.3)	11 (18.0)	

Continuous variables were expressed as means ± SD, while categorical variables were presented as percentages.

Table 2 presents the distribution of study participants according to the NWAT scores. The average total NWAT score was 62.20 ± 2.9, reflecting a moderate overall level of well-being among the nurses. When ranking the NWAT domains from lowest to highest scores, the order was: (1) work-life balance, (2) emotional and mental health, (3) work environment and support, (4) workload and burnout, (5) professional fulfillment, and (6) physical well-being. These findings emphasize critical areas needing attention, particularly work-life balance and emotional and mental health, followed by workload management, workplace support, and opportunities for professional growth and physical well-being.

**Table 2:** Distribution of the study participants according to the nurse’s well-being assessment tool (NWAT), (*n* = 169).

Questions	Strongly disagree *N* (%)	Disagree *N* (%)	Neutral *N* (%)	Agree *N* (%)	Strongly agree *N* (%)	Mean ± SD
1. Physical well-being						
1. I get adequate sleep most nights.	3.0 (1.8)	28 (16.6)	0.0 (0.0)	65 (38.5)	73 (43.2)	4.0 ± 1.1
2. I feel physically healthy and energized during my shifts.	0.0 (0.0)	13 (7.7)	9.0 (5.3)	38 (22.5)	109 (64.5)	4.4 ± 0.9
3. I have time to eat balanced meals during workdays.	19 (11.2)	10 (5.9)	20 (11.8)	88 (52.1)	32 (18.9)	3.6 ± 1.1
Total score (Mean ± SD): 12.10 ± 1.65				Rank from low to high: 6		
2. Emotional and mental health						
1. I feel emotionally supported in my role.	10 (5.9)	31 (18.3)	42 (24.9)	64 (37.9)	22 (13.0)	3.3 ± 1.1
2. I am able to manage stress effectively.	4.0 (2.4)	6.0 (3.6)	53 (31.4)	84 (49.7)	22 (13.0)	3.6 ± 0.8
3. I have access to mental health resources when needed.	24 (14.2)	65 (38.5)	38 (22.5)	18 (10.7)	24 (14.2)	2.7 ± 1.2
Total score (Mean ± SD): 9.73 ± 1.8				Rank from low to high: 2		
3. Workload and burnout						
1. I have a manageable workload.	13 (7.7)	14 (8.3)	23 (13.6)	69 (40.8)	50 (29.6)	3.7 ± 1.1
2. I feel overwhelmed by my responsibilities.	0.0 (0.0)	24 (14.2)	40 (23.7)	61 (36.1)	44 (26.0)	3.7 ± 1.0
3. I feel emotionally exhausted after work.	0.0 (0.0)	21 (12.4)	78 (46.2)	29 (17.2)	41 (24.3)	3.5 ± 0.9
Total score (Mean ± SD): 11.0 ± 2.4				Rank from low to high: 4		
4. Work environment and support						
1. I feel respected by colleagues and supervisors.	0.0 (0.0)	0.0 (0.0)	19 (11.2)	101 (59.8)	49 (29.0)	4.1 ± 0.6
2. I receive constructive feedback and recognition.	63 (37.3)	70 (41.4)	31 (18.3)	5.0 (3.0)	0.0 (0.0)	1.8 ± 0.8
3. I feel safe in my work environment.	0.0 (0.0)	4.0 (2.4)	22 (13.0)	98 (58.0)	45 (26.6)	4.0 ± 0.6
Total score (Mean ± SD): 10.1 ± 1.0				Rank from low to high: 3		
5. Work-life balance						
1. I am able to separate work from my personal life.	86 (50.9)	54 (32.0)	21 (12.4)	8.0 (4.7)	0.0 (0.0)	1.7 ± 0.8
2. I have enough time for family, hobbies, and self-care.	0.0 (0.0)	15 (8.9)	50 (29.6)	104 (61.5)	0.0 (0.0)	3.5 ± 0.6
3. I rarely have to work beyond my scheduled hours.	31 (18.3)	55 (32.5)	55 (32.5)	28 (16.6)	0.0 (0.0)	2.4 ± 0.9
Total score (Mean ± SD): 7.71 ± 1.2				Rank from low to high: 1		
6. Professional fulfillment						
1. I feel proud of the care I provide.	0.0 (0.0)	0.0 (0.0)	6.0 (3.6)	96 (56.8)	67 (39.6)	4.3 ± 0.5
2. My work gives me a sense of purpose.	0.0 (0.0)	26 (15.4)	28 (16.6)	89 (52.7)	26 (15.4)	3.6 ± 0.9
3. I feel supported in my professional development.	0.0 (0.0)	41 (24.3)	46 (27.2)	48 (28.4)	34 (20.1)	3.4 ± 1.0
Total score (Mean ± SD): 11.4 ± 1.5				Rank from low to high: 5		
Total Score of the NWAT:						62.20 ± 2.9

Continuous variables were expressed as means ± SD, while categorical variables were presented as percentages. The rank of the domains was according to the mean of the total scores for each domain.

Table 3 shows the distribution of study participants based on the WHO-5 Well-Being Index. The results indicated a mean total score of 12.70 ± 2.2, reflecting a low level of emotional well-being among the nurses. This highlights the necessity for targeted interventions to support the mental health and emotional needs of the nursing staff.

**Table 3:** Distribution of the study participants according to the world health organization-5 well-being index, (*n* = 169).

Questions	At no time *N* (%)	Some of the time *N* (%)	Less than half of the time *N* (%)	More than half of the time *N* (%)	Most of the time *N* (%)	All of the time *N* (%)	Mean ± SD
1. I have felt cheerful and in good spirits.	0.0 (0.0)	39 (23.1)	56 (33.1)	55 (32.5)	19 (11.2)	0.0 (0.0)	2.3 ± 0.9
2. I have felt calm and relaxed.	3.0 (1.8)	46 (27.2)	60 (35.5)	60 (35.5)	0.0 (0.0)	0.0 (0.0)	2.0 ± 0.8
3. I have felt active and vigorous.	6.0 (3.6)	72 (42.6)	54 (32.0)	24 (14.2)	13 (7.7)	0.0 (0.0)	1.7 ± 0.9
4. I woke up feeling fresh and rested.	0.0 (0.0)	7.0 (4.1)	27 (16.0)	69 (40.8)	48 (28.4)	18 (10.7)	3.2 ± 0.9
5. My daily life has been filled with things that interest me.	0.0 (0.0)	5.0 (3.0)	9.0 (5.3)	92 (54.4)	59 (34.9)	4.0 (2.4)	3.2 ± 0.7
Total Score of the WHO-5 Well-Being Index							12.70 ± 2.2

Continuous variables were expressed as means ± SD, while categorical variables were presented as percentages.

The QNWL survey results showed that nurses at Alsalam and Al Haram hospitals had a mean total score of 91.02 ± 6.0, reflecting a generally poor quality of work life. The domain scores ranked from lowest to highest were: (1) work-life balance, (2) managerial support, (3) nurse-physician relationship, (4) communication and interaction, (5) autonomy and professional fulfillment, and (6) workload and work stress. These results point to critical areas requiring improvement, with work-life balance and managerial support as the top priorities. Subsequent efforts should focus on enhancing nurse-physician relationships, communication, autonomy, professional fulfillment, and reducing workload and work-related stress (Table 4).

**Table 4:** Distribution of the study participants according to the quality of nursing work life (QNWL) scale, (*n* = 169).

Questions	Strongly disagree *N* (%)	Disagree *N* (%)	Neutral *N* (%)	Agree *N* (%)	Strongly agree *N* (%)	Mean ± SD
1. Work-life balance						
1. I feel I have a good balance between my work and personal life.	0.0 (0.0)	6.0 (3.6)	49 (29.0)	84 (49.7)	30 (17.8)	3.8 ± 0.7
2. I am able to fulfill my family and personal obligations despite my work demands.	0.0 (0.0)	19 (11.2)	43 (25.4)	58 (34.3)	49 (29.0)	3.8 ± 0.9
3. The hospital provides adequate time off for me to maintain my personal life outside of work.	16 (9.5)	77 (45.6)	69 (40.8)	7.0 (4.1)	0.0 (0.0)	2.3 ± 0.7
Total score (Mean ± SD): 10.0 ± 1.5				Rank from low to high: 1		
2. Nurse-physician relationship						
1. I feel respected by physicians in my workplace.	0.0 (0.0)	0.0 (0.0)	32 (18.9)	57 (33.7)	80 (47.3)	4.2 ± 0.7
2. There is effective communication between nurses and physicians in our hospital.	0.0 (0.0)	6.0 (3.6)	40 (23.7)	71 (42.0)	52 (30.8)	4.0 ± 0.8
3. I feel comfortable approaching physicians to discuss patient care or concerns.	46 (27.2)	88 (52.1)	27 (16.0)	8.0 (4.7)	0.0 (0.0)	1.9 ± 0.7
4. The collaboration between nurses and physicians is generally positive and supportive.	0.0 (0.0)	0.0 (0.0)	27 (16.0)	96 (56.8)	46 (27.2)	4.1 ± 0.6
Total score (Mean ± SD): 14.3 ± 1.9				Rank from low to high: 3		
3. Managerial support						
1. My nursing supervisor is available and approachable when I need guidance.	0.0 (0.0)	52 (30.8)	60 (35.5)	57 (33.7)	0.0 (0.0)	3.0 ± 0.8
2. I feel supported by my nursing managers in handling stressful situations at work.	44 (26.0)	68 (40.2)	35 (20.7)	22 (13.0)	0.0 (0.0)	2.2 ± 0.9
3. My nursing supervisor provides me with constructive feedback on my performance.	0.0 (0.0)	64 (37.9)	88 (52.1)	17 (10.1)	0.0 (0.0)	2.7 ± 0.6
4. I feel that my contributions to patient care are valued by my manager.	17 (10.1)	59 (34.9)	57 (33.7)	36 (21.3)	0.0 (0.0)	2.6 ± 0.9
5. I receive adequate support from management when dealing with difficult patients or families.	0.0 (0.0)	23 (13.6)	65 (38.5)	81 (47.9)	0.0 (0.0)	3.3 ± 0.7
Total score (Mean ± SD): 13.9 ± 2.1				Rank from low to high: 2		
4. Communication and interaction						
1. I feel well-informed about changes in hospital policies and procedures.	0.0 (0.0)	3.0 (1.8)	31 (18.3)	69 (40.8)	66 (39.1)	4.1 ± 0.7
2. There is clear communication between nurses in my department.	0.0 (0.0)	0.0 (0.0)	23 (13.6)	70 (41.4)	76 (45.0)	4.3 ± 0.7
3. I am encouraged to share my ideas and suggestions with my colleagues and managers.	0.0 (0.0)	18 (10.7)	46 (27.2)	60 (35.5)	45 (26.6)	3.7 ± 0.9
4. The nursing team works well together to solve problems and improve patient care.	0.0 (0.0)	0.0 (0.0)	28 (16.6)	120 (71.0)	21 (12.4)	3.9 ± 0.5
Total score (Mean ± SD): 16.2 ± 1.8				Rank from low to high: 4		
5. Workload and work stress						
1. My workload is manageable given the time and resources available.	0.0 (0.0)	20 (11.8)	58 (34.3)	44 (26.0)	47 (27.8)	3.6 ± 1.0
2. The hospital is adequately staffed to meet patient care needs.	10 (5.9)	71 (42.0)	76 (45)	12 (7.1)	0.0 (0.0)	2.5 ± 0.7
3. I frequently feel overwhelmed by my workload.	0.0 (0.0)	76 (45.0)	72 (42.6)	21 (12.4)	0.0 (0.0)	2.6 ± 0.6
4. I feel that the expectations placed on me at work are realistic.	0.0 (0.0)	19 (11.2)	64 (37.9)	86 (50.9)	0.0 (0.0)	3.3 ± 0.6
5. The demands of my work leave me feeling physically exhausted by the end of my shift.	0.0 (0.0)	0.0 (0.0)	52 (30.8)	86 (50.9)	31 (18.3)	3.8 ± 0.6
6. I often feel stressed due to the pace of work and patient care responsibilities.	16 (9.5)	90 (53.3)	53 (31.4)	10 (5.9)	0.0 (0.0)	2.3 ± 0.7
Total score (Mean ± SD): 18.5 ± 1.8				Rank from low to high: 6		
6. Autonomy and professional fulfillment						
1. I have a sense of autonomy in making decisions about patient care.	0.0 (0.0)	43 (25.4)	59 (34.9)	67 (39.6)	0.0 (0.0)	3.1 ± 0.7
2. I feel that I have enough freedom in my role to make decisions that affect my patients’ health.	66 (39.1)	75 (44.4)	22 (13.0)	6.0 (3.6)	0.0 (0.0)	1.8 ± 0.7
3. My work as a nurse is personally fulfilling.	0.0 (0.0)	64 (37.9)	70 (41.4)	27 (16.0)	8.0 (4.7)	2.8 ± 0.8
4. I feel that my nursing role allows me to use my skills and knowledge to the fullest extent.	3.0 (1.8)	19 (11.2)	87 (51.5)	50 (29.6)	10 (5.9)	3.2 ± 0.8
5. I have opportunities for professional growth and development in my nursing career.	0.0 (0.0)	32 (18.9)	77 (45.6)	46 (27.2)	14 (8.3)	3.2 ± 0.8
6. I feel a sense of accomplishment in my nursing work.	8.0 (4.7)	14 (8.3)	38 (22.5)	91 (53.8)	18 (10.7)	3.5 ± 0.9
Total score (Mean ± SD): 17.9 ± 2.8				Rank from low to high: 5		
The QNWL Scale Total Score:						91.02 ± 6.0

Continuous variables were expressed as means ± SD, while categorical variables were presented as percentages. The rank of the domains was according to the mean of the total scores for each domain.

Table 5 shows the relationships between nurses’ well-being, measured by the NWAT and the WHO-5 well-being index, and their total QNWL scores. The analysis found a statistically significant correlation between overall NWAT scores and QNWL scores (*p* = 0.001), indicating that higher levels of well-being are associated with better perceived quality of work life. Similarly, the WHO-5 well-being index scores were also significantly linked to QNWL scores (*p* = 0.001), suggesting that emotional well-being alone can be a strong predictor of the overall quality of nursing work life.

**Table 5:** Associations between the nurses’ well-being total scores and the WHO well-being index total scores with the total score of quality of nursing work life, (*n* = 169).

Variables		Sum of Squares	Df	Mean Square	*F*	*p* value
The nurses’ well-being total scores	Between Groups	688.599	22	31.300	5.737	0.001
	Within Groups	796.561	146	5.456		
	Total	1485.160	168			
The WHO well-being index total scores	Between Groups	228.358	22	10.380	2.457	0.001
	Within Groups	616.849	146	4.225		
	Total	845.207	168			

The differences between means were tested using One-Way ANOVA. A *p*-value of less than 0.05 was considered statistically significant.

Table 6 illustrates that nurses at Alsalam Hospital scored higher on the total QNWL Scale and the WHO Well-Being Index but had lower total scores on the Nurses’ Well-Being Assessment Tool compared to nurses at Al Haram Hospital. Additionally, statistically significant differences were observed between the two hospitals in both the WHO Well-Being Index and QNWL survey total scores (*p*-values < 0.05).

**Table 6:** The nurses’ well-being total scores, the WHO well-being index total scores, and the quality of nursing work life survey total scores by hospitals, (*n* = 169).

Variables	Mean ± SD	*p*-value
Total score of the quality of nursing work life (QNWL)		
Alsalam hospital	92.21 ± 6.5	0.001
Al Haram hospitals	88.91 ± 4.5	
The nurses’ well-being total scores		
Alsalam hospital	61.57 ± 3.0	0.001
Al Haram hospitals	63.31 ± 2.5	
The World Health Organization (WHO) well-being index total scores		
Alsalam hospital	12.96 ± 2.4	0.046
Al Haram hospitals	12.24 ± 1.6	

Continuous variables were expressed as means ± SD. The differences between means were tested using Independent-Samples T Test. A *p*-value of less than 0.05 was considered statistically significant.

## 4 Discussion

This study aimed to evaluate the well-being of nurses at Alsalam and Al Haram hospitals in Saudi Arabia and examine how it relates to the QNWL. The results reveal important insights into the physical, emotional, and professional challenges nurses face, highlighting areas that require urgent intervention to enhance both nurse well-being and healthcare quality.

### 4.1 Nurses’ well-being and emotional health

The NWAT results showed a moderate overall well-being score, indicating that nurses at the two hospitals experience a mix of both positive and negative well-being factors. The domains with the lowest scores were work-life balance and emotional and mental health, pinpointing these as critical stress points. This finding resonates with extensive literature indicating that nurses worldwide often struggle to maintain a healthy balance between demanding work schedules and personal lives. For instance, previous studies (21, 22) showed similar challenges among nurses in Saudi Arabia, where long shifts, staffing shortages, and high patient acuity contribute to work-life conflict and emotional exhaustion.

The WHO-5 Well-Being Index further underscored a concerning low emotional well-being level with a mean score of 12.70 ± 2.2, below the threshold indicating good mental health. This finding aligns with prior studies in both regional and global contexts where nurses frequently report symptoms of depression, anxiety, and burnout (23, 24). The emotional strain documented here is likely influenced by factors such as high workloads, workplace stress, and limited psychosocial support. These low well-being scores emphasize the urgent need for targeted mental health support initiatives within these hospitals. Similar stress-related factors have been reported among healthcare students, indicating that stress begins early in professional development and may persist into clinical practice (25). Previous research on frontline healthcare workers during the COVID-19 pandemic also highlighted significant emotional burden, poor quality of life, and the long-term psychological toll of high-intensity clinical settings (26).

### 4.2 Quality of nursing work life (QNWL)

The QNWL scale revealed an overall poor quality of work life at Alsalam and Al Haram hospitals, with a mean score of 91.02 ± 6.0, which falls below the moderate threshold. The lowest scoring domains were work-life balance and managerial support, indicating that nurses feel unsupported by management and unable to reconcile job demands with personal life. This is consistent with previous studies findings (27, 28) which noted that poor managerial support significantly diminishes nurse satisfaction and increases turnover intention. Despite workload and work stress scoring relatively higher, the overall low QNWL score suggests that nurses may be coping with their workload but are hampered by organizational and interpersonal shortcomings.

Notably, nurse-physician relationships, communication, autonomy, and professional fulfillment scored moderately, signaling some positive aspects of the work environment. However, these factors alone were insufficient to counterbalance the detrimental effects of poor work-life integration and weak managerial support. This imbalance may also reflect systemic issues in hospital culture and leadership that fail to prioritize nurse well-being as a strategic objective.

### 4.3 Relationship between well-being and quality of work life

Statistical analysis showed significant positive correlations between NWAT and WHO-5 scores with QNWL scores, indicating that higher nurse well-being, both physical and emotional, is closely associated with better perceptions of work life quality. This relationship mirrors findings from previous studies (29, 30) which emphasized that nurse well-being is a key predictor of job satisfaction, retention, and professional commitment. These results strongly support the hypothesis that improving nurse well-being is not only a moral imperative but also an organizational necessity to enhance workforce stability and patient care quality.

The interdependency between well-being and QNWL found in this study suggests that interventions aimed at reducing burnout and enhancing mental health may directly impact nurses’ overall work satisfaction. This highlights the need for healthcare institutions to adopt a holistic approach that addresses physical health, emotional resilience, workplace culture, and leadership support simultaneously. Psychological support mechanisms such as accessible counseling services have shown potential in mitigating burnout and improving work satisfaction (31).

### 4.4 Differences between Alsalam and Al Haram hospitals

Comparative analysis revealed an intriguing contrast: nurses at Alsalam Hospital scored higher on the QNWL scale and WHO-5 index but lower on the NWAT compared to Al Haram Hospital nurses. These discrepancies may reflect differences in hospital policies, resource allocation, leadership effectiveness, and workforce demographics. Alsalam Hospital, being larger and perhaps more established, may offer better organizational support structures and professional development opportunities, thereby improving work life quality and emotional well-being. Conversely, the lower NWAT score at Alsalam could suggest that while nurses feel supported and satisfied at work, physical well-being or other personal health factors might be more compromised. These findings underscore the importance of context-specific assessments when designing workplace interventions. Policies and programs effective in one hospital may not translate seamlessly to another due to differences in culture, staff composition, and operational challenges.

The results indicate that nurses in these Saudi Arabian hospitals face multidimensional well-being challenges with clear consequences for their quality of work life. The moderate to low well-being and poor QNWL scores suggest systemic issues related to workload management, leadership engagement, and emotional support mechanisms. Given Saudi Arabia’s ambitious Vision 2030 healthcare goals, which emphasize service excellence and workforce sustainability, addressing nurse well-being must be prioritized alongside infrastructural and technological upgrades. The study’s findings contribute to the growing evidence that well-being is a cornerstone of nursing workforce stability and healthcare quality. Hospitals that fail to invest in nurses’ mental, emotional, and physical health risk higher turnover, absenteeism, and compromised patient care. The multicultural nature of the nursing staff, especially with a significant expatriate workforce, adds complexity requiring culturally sensitive support systems and inclusive workplace policies. However, this study provides limited description and analysis of expatriate nurses’ characteristics, as detailed data on nationality and cultural background were not collected. This gap restricts a deeper understanding of how cultural and regulatory challenges uniquely affect expatriate nurses’ well-being and quality of work life. Future research should focus on these aspects to develop targeted support strategies for this important subgroup. Expatriate nurses may face additional stressors related to cultural adjustment, communication barriers, and regulatory constraints, which can exacerbate emotional exhaustion and work-life imbalance. Culturally tailored support programs and inclusive workplace policies are crucial to addressing these issues and fostering a supportive environment for all nurses. Integrating cultural competence into leadership training and well-being initiatives could improve nurse retention and satisfaction, ultimately enhancing patient care quality.

Finally, to improve nurse well-being and quality of work life, it is important to consider the potential development and implementation of tailored mental health support programs, including accessible counseling and stress reduction workshops aimed at addressing emotional exhaustion and building resilience. Evidence indicates that psychological interventions such as mindfulness-based stress reduction and cognitive-behavioral strategies can significantly reduce burnout and improve mental health among nurses (32). Accessible counseling services have also been shown to promote emotional resilience and job satisfaction in healthcare settings (33). Enhancing managerial training focused on supportive leadership, effective communication, and conflict resolution may strengthen the perceived managerial support among nursing staff. Studies suggest that transformational leadership and manager training positively influence nurse retention and work satisfaction by fostering a supportive and empowering work environment (34, 35). Promoting work-life balance through flexible scheduling, limiting overtime, and encouraging adequate rest is a practical approach to help nurses manage professional and personal demands. Research supports that flexible work arrangements and policies that respect nurses’ personal time reduce stress and improve work-life integration (36).

Strengthening nurse-physician collaboration by facilitating regular interprofessional meetings and team-building activities could improve workplace relationships and communication, which are key factors in enhancing nurse job satisfaction and patient outcomes. Interprofessional collaboration initiatives have been linked to better communication, teamwork, and reduced workplace conflict (37). Given the diversity of the nursing workforce, cultural competence programs that include orientation and ongoing inclusivity training might help reduce social isolation and foster better teamwork. Incorporating cultural competence into leadership and staff development has been linked to improved workplace climate and nurse retention (38). Additionally, routine monitoring and evaluation of well-being and QNWL metrics are recommended to support identification of trends and assessment of interventions for continuous improvement. Finally, integrating these initiatives with Saudi Arabia’s Vision 2030 healthcare reform may facilitate sustained funding, strategic alignment, and high-level support for workforce sustainability.

### 4.5 Strengths and limitations

This study’s major strength lies in its thorough assessment of nurses’ well-being and quality of work life at Alsalam and Al Haram hospitals, using validated instruments like the NWAT, WHO-5, and QNWL scale. Employing a census sampling method that included all eligible nurses enhances the reliability and generalizability of the findings within these institutions. The study provides valuable insights into factors influencing nurse well-being, such as workload, emotional exhaustion, and workplace support, which can inform policy and management strategies. However, the cross-sectional design limits the ability to establish causality, and the focus on public hospitals in a single region restricts the applicability of results to other healthcare settings, such as private institutions. The study did not consider external personal stressors that may affect nurse well-being. While Chi-square tests were used to compare baseline demographics between the two hospitals, these comparisons were not central to the study objectives and may offer limited meaningful information. Future research should employ stratified analyses to examine hospital-based differences in outcomes more effectively. Furthermore, the inability to analyze data by nationality or cultural background highlights a limitation, emphasizing the need for future studies to investigate how the complex dynamics of a multicultural and migrant nursing workforce influence nurses’ experiences and outcomes. Additionally, regarding the relevance to nurses’ quality of life, while gender-related social roles may influence work-life balance and well-being, our study was limited to biological sex as a demographic variable. Future research could benefit from exploring gender identity and roles more explicitly to capture their potential impact on nurse well-being and quality of work life.

## 5 Conclusion

This study highlights the pressing need to address nurse well-being comprehensively to improve the quality of nursing work life at Alsalam and Al Haram hospitals. The strong correlation between well-being and QNWL underscores that interventions must target both physical and emotional health, alongside organizational and cultural improvements. By doing so, hospitals can build a healthier, more satisfied nursing workforce that is better equipped to meet the demands of Saudi Arabia’s evolving healthcare system, ultimately advancing patient care quality and supporting Vision 2030’s objectives.

## Data Availability

The raw data supporting the conclusions of this article will be made available by the authors, without undue reservation.

## References

[1] Kruger K Brysiewicz P Lori J Bell SA. The role of the nursing workforce in health system resilience during disasters: a scoping review of empirical studies. Int J Nurs Stud Adv. (2025) 9:100361. doi: 10.1016/j.ijnsa.2025.100361, PMID: 40600236 PMC12210316

[2] World Health Organization. WHO guidelines on mental health at work. Geneva: World Health Organization (2022).

[3] Kailay M Paposa KK Chhibber P. Challenges and motivators for nurses' well-being during and post-COVID-19 pandemic: a qualitative exploration. TQM J. (2025) 37:778–99. doi: 10.1108/TQM-07-2023-0229

[4] Rahman R Al-Borie HM. Strengthening the Saudi Arabian healthcare system: role of vision 2030. Int J Health Care Manag. (2021) 14:1483–91. doi: 10.1080/20479700.2020.1788334

[5] Aldhafeeri MS Alharbi AFT Aldhafeeri BHM Aldhafeeri FH Alshammari SS Alshammari MH. The role of health assistants in implementing Saudi vision 2030 healthcare goals: a systematic review of current evidence. J Int Crisis Risk Commun Res. (2024) 7:2640. doi: 10.63278/jicrcr.vi.1971

[6] Alenezi A Alhowaymel FM Abaoud AF Mostafa MH. The relationship between psychological capital and humanistic caring ability among mental health nurses in Saudi Arabia. BMC Nurs. (2024) 23:688. doi: 10.1186/s12912-024-02344-7, PMID: 39334372 PMC11438261

[7] Alotaibi K Higgins I Chan S. Culture, religion, language and the assessment and management of children's pain by expatriate nurses in Saudi Arabia: a qualitative study. J Spec Pediatr Nurs. (2023) 28:e12399. doi: 10.1111/jspn.12399, PMID: 36419370

[8] Robertson IT Cooper CL Johnson S. Well-being: Productivity and happiness at work. Basingstoke: Palgrave Macmillan (2011).

[9] Al Menji H Al Zadjali A Al Lamki M Al Shamsi Z Al Kiyumi L Al Harrasi K. Health system and absenteeism among nurses in Oman: root cause analysis. J Oman Med Assoc. (2024) 1:48–60. doi: 10.3390/joma1010006

[10] Brooks BA Anderson MA. Defining quality of nursing work life. Nurs Econ. (2005) 23:316–20. 16459904

[11] Alshammari B Alshammari MM Baghdadi NA. The association between missed nursing care and job satisfaction among nurses in Saudi Arabian hospitals: a cross-sectional study. Nurs Rep. (2025) 15:296. doi: 10.3390/nursrep15080296, PMID: 40863683 PMC12389183

[12] Giordano NA Razmpour O Mascaro JS Kaplan DM Lewis AS Baird M. Reliability and validity of measures commonly utilized to assess nurse well-being. Nurs Res. (2024) 73:399–405. doi: 10.1097/NNR.0000000000000752 38842438

[13] de Souza CM Hidalgo MPL. World Health Organization 5-item well-being index: validation of the Brazilian Portuguese version. Eur Arch Psychiatry Clin Neurosci. (2012) 262:239–44. doi: 10.1007/s00406-011-0255-x, PMID: 21912931

[14] Brooks BA Anderson MA. Defining quality of nursing work life. Nurs Econ. (2005) 23:319–26. PMID: 16459904

[15] Tamimi BA Melhem L Alhoor R Qabajah R. (2017). Structural Design of Al-Salam Hospital. Palestine, Polytechnic University. Available at: https://scholar.ppu.edu/bitstream/handle/123456789/6061/Structural%20Desighn%20of%20Al-Salam%20Hospital.pdf?sequence=2&isAllowed=y (Accessed on July, 2025).

[16] Wilson RT Hasanali SH Sheikh M Cramer S Weinberg G Firth A. Challenges to the census: international trends and a need to consider public health benefits. Public Health. (2017) 151:87–97. doi: 10.1016/j.puhe.2017.05.015, PMID: 28759883

[17] Al Mutair A Al Bazroun MI Almusalami EM Aljarameez F Alhasawi AI Alahmed F. Quality of nursing work life among nurses in Saudi Arabia: a descriptive cross-sectional study. Nurs Rep. (2022) 12:1014–22. doi: 10.3390/nursrep12040097, PMID: 36548170 PMC9783332

[18] Suleiman K Hijazi Z Al Kalaldeh M Abu Sharour L. Quality of nursing work life and related factors among emergency nurses in Jordan. J Occup Health. (2019) 61:398–406. doi: 10.1002/1348-9585.12068, PMID: 31215754 PMC6718837

[19] Nylén-Eriksen M Bjørnnes AK Hafstad H Lie I Grov EK Lara-Cabrera ML. Validating the five-item World Health Organization well-being index. Int J Environ Res Public Health. (2022) 19:11489. doi: 10.3390/ijerph191811489, PMID: 36141760 PMC9517039

[20] Lara-Cabrera ML Betancort M Muñoz-Rubilar A Rodríguez-Novo N Bjerkeset O Cuevas CDL. Psychometric properties of the WHO-5 well-being index among nurses during the COVID-19 pandemic: a cross-sectional study in three countries. Int J Environ Res Public Health. (2022) 19:10106. doi: 10.3390/ijerph191610106, PMID: 36011741 PMC9407690

[21] Al Yahyaei A Rabaani EA Alkasbi R Alhashmi Y Hatmi IA. Examining the influence of shift length on nurse fatigue, patient care, quality of life, and work-life dynamics in a tertiary Hospital in Oman: comparative study. Nurs Res Pract. (2025) 2025:7946997. doi: 10.1155/nrp/7946997, PMID: 40662080 PMC12259321

[22] Jaber MJ Bindahmsh AA Baker OG Alaqlan A Almotairi SM Elmohandis ZE. Burnout combating strategies, triggers, implications, and self-coping mechanisms among nurses working in Saudi Arabia: a multicenter, mixed methods study. BMC Nurs. (2025) 24:590. doi: 10.1186/s12912-025-03191-w, PMID: 40420210 PMC12107983

[23] Sischka PE Martin G Residori C Hammami N Page N Schnohr C. Cross-national validation of the WHO-5 well-being index within adolescent populations: findings from 43 countries. Assessment. (2025) 10731911241309452. doi: 10.1177/10731911241309452 39884717

[24] Domenech A Kasujee I Koscielny V Griffiths CE. Systematic review of the use of the WHO-5 well-being index across different disease areas. Adv Ther. (2025) 1–21. doi: 10.1007/s12325-025-03266-9 40506676 PMC12313798

[25] Ullah H Arbab S Liu CQ Du Q Khan SA Khan S. Source of stress-associated factors among medical and nursing students: a cross-sectional study. J Nurs Manag. (2025) 2025:9928649. doi: 10.1155/jonm/9928649, PMID: 40709104 PMC12289356

[26] Ullah H Arbab S Liu CQ Khan SA Shahzad S Li K. Professional quality of life and psychological impact on frontline healthcare worker during the fourth wave of COVID-19. J Nurs Manag. (2024) 2024:2865063. doi: 10.1155/2024/2865063 40224890 PMC11919194

[27] Modaresnezhad M Andrews MC Mesmer-Magnus J Viswesvaran C Deshpande S. Anxiety, job satisfaction, supervisor support and turnover intentions of mid-career nurses: a structural equation model analysis. J Nurs Manag. (2021) 29:931–42. doi: 10.1111/jonm.13229, PMID: 33617110

[28] Liu W Zhao S Shi L Zhang Z Liu X Li LI. Workplace violence, job satisfaction, burnout, perceived organisational support and their effects on turnover intention among Chinese nurses in tertiary hospitals: a cross-sectional study. BMJ Open. (2018) 8:e019525. doi: 10.1136/bmjopen-2017-019525, PMID: 29886440 PMC6009508

[29] AlMuzaini AS AlRasheedi BS AlShahrani FF AlOtaibi HB AlHagan JAM AlOtaibi KRH. Workplace well-being in nursing: a systematic review of trends in burnout, job satisfaction, and retention. J Int Crisis Risk Commun Res. (2024) 7:3103. doi: 10.63278/jicrcr.vi.2174

[30] Galletta M Portoghese I Coppola RC Finco G Campagna M. Nurses well-being in intensive care units: study of factors promoting team commitment. Nurs Crit Care. (2016) 21:146–56. doi: 10.1111/nicc.12083, PMID: 24750240

[31] Ullah H Arbab S Khan SA Liu CQ Fayaz M Tian Y. Work-related stress, professional respect, and psychological counseling among nurses: a cross-sectional study. J Nurs Manag. (2025) 2025:2413658. doi: 10.1155/jonm/2413658, PMID: 40709105 PMC12289370

[32] Sulosaari V Unal E Cinar FI. The effectiveness of mindfulness-based interventions on the psychological well-being of nurses: a systematic review. Appl Nurs Res. (2022) 64:151565. doi: 10.1016/j.apnr.2022.151565, PMID: 35307128

[33] Kyambade M Namatovu A. Pleasurable emotional states in health-care organizations: the mediation role of employee wellbeing on transformational leadership and job satisfaction. Leadersh Health Serv. (2025) 38:299–317. doi: 10.1108/LHS-06-2024-0052, PMID: 39887242

[34] Goens B Giannotti N. Transformational leadership and nursing retention: an integrative review. Nurs Res Pract. (2024) 2024:3179141. doi: 10.1155/2024/3179141, PMID: 39070779 PMC11283332

[35] Ystaas LMK Nikitara M Ghobrial S Latzourakis E Polychronis G Constantinou CS. The impact of transformational leadership in the nursing work environment and patients’ outcomes: a systematic review. Nurs Rep. (2023) 13:1271–90. doi: 10.3390/nursrep13030108, PMID: 37755351 PMC10537672

[36] Ingstad K Haugan G. Balancing act: exploring work-life balance among nursing home staff working long shifts. BMC Nurs. (2024) 23:499. doi: 10.1186/s12912-024-02165-8, PMID: 39039590 PMC11264412

[37] Cleary M Foong A Kornhaber R McLean L Visentin DC. Interprofessional collaborations for improved health care. Issues Ment Health Nurs. (2019) 40:1045–8. doi: 10.1080/01612840.2019.1655367, PMID: 31693424

[38] King R Taylor B Talpur A Jackson C Manley K Ashby N. Factors that optimise the impact of continuing professional development in nursing: a rapid evidence review. Nurse Educ Today. (2021) 98:104652. doi: 10.1016/j.nedt.2020.104652, PMID: 33190952

